# 16S rRNA gene amplicon sequencing data of microbial communities associated with methane concentrations in sediment microbial fuel cell systems

**DOI:** 10.1128/mra.00802-26

**Published:** 2026-09-04

**Authors:** Yasuyuki Takemura, Masataka Aoki, Keiichi Kubota, Kazuaki Syutsubo

**Affiliations:** 1 Regional Environment Conservation Division, National Institute for Environmental Studies, Ibaraki, Japan; 2 Department of Materials and Environment, Graduate School of Science and Technology, Gunma University, Gunma, Japan; 3 Research Center for Water Environment Technology, School of Engineering, The University of Tokyo, Tokyo, Japan; USDA-ARS San Joaquin Valley Agricultural Sciences Center, Parlier, California, USA

**Keywords:** sediment microbial fuel cells, methane concentration, *Methanoperedenaceae*

## Abstract

Sediment microbial fuel cells (SMFCs) were constructed using different sediments, and microbial communities were analyzed by 16S rRNA gene amplicon sequencing. Methane concentrations were lower in roadside ditch sediment under SMFC conditions than under control conditions. *Methanoperedenaceae* was selectively enriched under SMFC conditions, suggesting a potential role in methane oxidation.

## ANNOUNCEMENT

Sediment microbial fuel cells (SMFCs) are promising systems for energy generation and environmental remediation (1), but their effects on methane behavior remain unclear (2). Here, we present methane concentrations in the headspace and 16S rRNA gene amplicon profiles of sediment microbiota in SMFC systems.

In this study, four types of environmental samples (artificial pond sediment [A], roadside ditch sediment [B], vegetated soil [C], and wet soil [D]) were used to construct laboratory-scale SMFC systems (Table 1). These sediments were placed into cylindrical columns (sediment depth, 10 cm; overlying water, 20 cm). Carbon felt was used as the electrode material (anode depth: 5 cm, cathode depth: 10 cm). The electrodes were fabricated as described in a previous study (3). For each sample type, duplicate columns were operated under closed-circuit (SMFC; 1 kΩ) and open-circuit (control) conditions for over 1 year to stabilize electricity generation. For methane concentration measurements, 1-L headspace gas bags filled with nitrogen were connected to the closed columns. The columns were incubated at 30°C in the dark. Methane concentrations were measured weekly during 48 days using headspace gas bags and gas chromatography. Methane concentrations in the headspace, as an indicator of methane emissions, were markedly higher in roadside ditch sediment (B) under control conditions (approximately 500–5,000 ppm). In contrast, methane concentrations were lower in this sediment under SMFC conditions and comparable to those in other sediments under both SMFC and control conditions (approximately 100–400 ppm).

**TABLE 1:** Profiles of sediment samples and 16S rRNA gene amplicons

Sample ID	Sediment type^*a*^	Electrical circuit conditions^*b*^	No. of raw reads	No. of ASVs^*c*^	F index (5′→3′)	R index (5′→3′)	SRA accession no.
A_initial	A	Initial	49,827	40,011	AGAGGTTACA	CAGAAGTTAG	SRR39313689
B_initial	B	Initial	49,429	35,629	CAGAAGTTAG	AGAGGTTACA	SRR39313688
C_initial	C	Initial	49,085	36,547	ACAACGCCAA	CTGGACATAA	SRR39313677
D_initial	D	Initial	48,833	36,599	CTGGACATAA	ACAACGCCAA	SRR39313676
A_control_1	A	OC	49,794	40,984	CCTCCTCTTG	AGAGCAGTAT	SRR39313675
A_control_2	A	OC	49,961	40,716	CGCTTCACAG	TTAGGTGAAG	SRR39313674
A_SMFC_1	A	CC	49,008	40,692	TTAGGTGAAG	CGCTTCACAG	SRR39313673
A_SMFC_2	A	CC	49,074	40,320	TTGTACTCGC	ATTACGCCGC	SRR39313672
B_control_1	B	OC	49,702	37,386	AACTCCAAGA	GCCTTCACAT	SRR39313671
B_control_2	B	OC	49,529	36,980	GCCTTCACAT	AACTCCAAGA	SRR39313670
B_SMFC_1	B	CC	49,688	36,830	ATCCAGGTTA	ATGCTCTCTT	SRR39313687
B_SMFC_2	B	CC	48,558	36,855	ATGCTCTCTT	ATCCAGGTTA	SRR39313686
C_control_1	C	OC	48,996	38,861	AAGTGATCCA	GAGTTGAGGC	SRR39313685
C_control_2	C	OC	49,433	39,111	AGGCCTCTTC	ATCTGACGGA	SRR39313684
C_SMFC_1	C	CC	49,820	38,860	ATCTGACGGA	AGGCCTCTTC	SRR39313683
C_SMFC_2	C	CC	48,870	39,043	GAGTTGAGGC	AAGTGATCCA	SRR39313682
D_control_1	D	OC	49,595	39,233	AGGCCTTATA	CAACCAGCGA	SRR39313681
D_control_2	D	OC	50,009	39,633	CAACCAGCGA	AGGCCTTATA	SRR39313680
D_SMFC_1	D	CC	48,649	38,369	GTTCCACCGA	TAACAGCAGG	SRR39313679
D_SMFC_2	D	CC	48,604	37,908	TAACAGCAGG	GTTCCACCGA	SRR39313678

^*a*^ A, artificial pond sediment (algal detritus-rich, flocculent structure); B, roadside ditch sediment (fine-grained, water-saturated mud); C, vegetated soil (surface soil densely covered with herbaceous vegetation); and D, wet soil (waterlogged soil collected from a poorly drained area). All samples were collected within the campus of the National Institute of Technology (KOSEN), Wakayama College, Wakayama, Japan (33.50° N, 135.10° E) in November 2024. Samples used for initial community analysis were stored at −20°C immediately after collection and subsequently freeze-dried prior to DNA extraction.

^*b*^ Initial, samples collected before SMFC column setup (baseline microbial communities); OC, open-circuit (as control); CC, closed-circuit (acts as SMFC).

^*c*^ ASV numbers are shown before removal of rare variants.

At the end of the experimental period, 0.5 g (wet weight) sediment samples adhering to the anode in 16 columns were collected (Table 1). Sediment samples were freeze-dried using a VD-250 lyophilizer (Taitec), subjected to DNA extraction using an ISOFECAL kit (Nippon Gene), and purified using a LabAid824s DNA extraction kit (Zeesan). The amplicon libraries of the V4 region of prokaryotic 16S rRNA genes were prepared using PCR with 515F and 806R primers (4). The libraries were sequenced at Bioengineering Lab Co., Ltd. using the NextSeq 1000 system (2 × 300 bp paired-end reads) with the NextSeq 1000 P1 XLEAP-SBS Reagent kit (Illumina). Sequence data were processed using QIIME 2 v2025.7 (5). Reads matching the primer sequences were extracted using FASTX-Toolkit v0.0.14 (6), and primer sequences were trimmed. Low-quality reads (quality score <20) were filtered using sickle v1.33 (7). Paired-end reads were merged using FLASH v1.2.11 (8) with a minimum overlap of 10 bp for V4 amplicons (≥250 bp). Amplicon sequence variants (ASVs) were generated using DADA2 (9), and taxonomic classification was assigned using the SILVA database v138.2 (10). Detailed methods are available on the Protocols.io platform (11).

**Fig 1:**
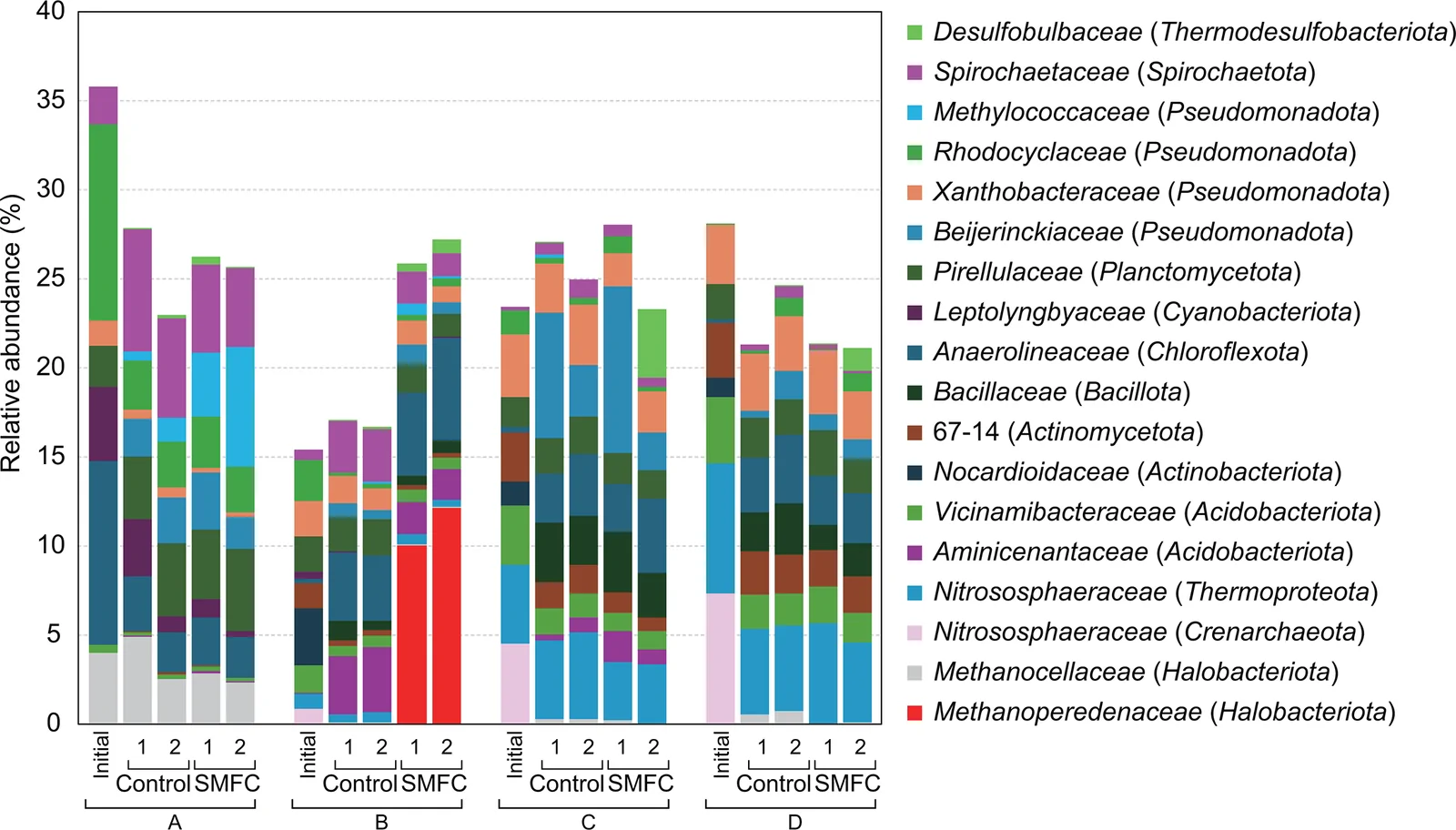
Relative abundance of prokaryotic (bacterial and archaeal) communities at the family level (more than 3% in at least one of the analyzed samples) in the samples adhering to the anode materials and initial sediments (baseline samples). The names in parentheses indicate the phylum-level classifications. A, artificial pond sediment; B, roadside ditch sediment; C, vegetated soil; D, wet soil. The figure was generated using Microsoft Excel.

*Methanoperedenaceae* was exclusively enriched in SMFC systems with roadside ditch sediment (B), reaching 10.0% and 12.2% in duplicate samples, while it was not detected in control samples and other sediment types (A, C, and D) (Fig. 1). Given the high methane concentrations observed in sediment B under control conditions, this finding suggests that *Methanoperedenaceae* may contribute to methane oxidation and potentially to lower methane concentrations under SMFC conditions, although potential effects of inoculum differences cannot be excluded.

## Data Availability

The sequences were submitted to the NCBI Sequence Read Archive (SRA) under the BioProject accession number PRJNA1481404. Individual SRA run accession numbers are provided in Table 1.
